# Empirical antibiotic use, resistance patterns, and their impact on clinical outcomes in a Yemeni tertiary hospital

**DOI:** 10.1016/j.nmni.2026.101723

**Published:** 2026-02-05

**Authors:** Adel Alshaikh, Mohammed Kubas

**Affiliations:** Lebanese International University, Department of Pharmacy and Medical Sciences, School of Clinical Pharmacy, Sana'a, Yemen

**Keywords:** Antibiotics, Resistance, Clinical outcomes, Empirical therapy, Susceptibility, Yemen, Antimicrobial stewardship

## Abstract

**Background:**

Antibiotic (AB) resistance is a global health threat, particularly in resource-limited settings like Yemen. Antibiotics misuse, especially in hospitals, is the most important risk for resistance development. Understanding empirical AB use and susceptibility patterns among inpatient settings is crucial for implementing effective antimicrobial stewardship.

**Objectives:**

To evaluate Empirical Antibiotic Therapy (EAT), resistance patterns, and their impact on hospitalized patients’ outcomes.

**Methods:**

A prospective cross-sectional study was conducted in medical and surgical wards in a tertiary hospital over 2 months, including 80 adult patients for analysis. The patient-level data on antibiotic prescriptions, culture results, patient demographics, clinical characteristics, and treatment outcomes, were collected manually and from computer records. An ethical approval was obtained, and SPSS app was used in analyzing the data.

**Results:**

EAT was alarmingly high (98%), often mismatched susceptibility patterns, coupled with high percentage (68.3%) of negative culture results. The most commonly prescribed antibiotics were Ceftriaxone, Vancomycin, Levofloxacin, Meropenem, Imipenem/Cilastatin, and Metronidazole. Significant resistance (>50%) was observed against Moxifloxacin, Clindamycin, and all tested beta-lactam agents except Cefuroxime (42%). Appropriate EAT was associated with good prognosis, *P* = .029 (Fisher's Exact Test), and Ceftriaxone use was associated with poorer prognosis, *P* = .017 (*X*^*2*^ Test).

**Conclusion:**

The study highlights a notable misuse of EAT coupled with high resistance rate in hospital. These findings underscore the need for effective interventions to optimize antibiotic use and mitigate the growing threat of resistance in Yemen. Strategies such as enhanced diagnostic capabilities, improved stewardship programs, and rational prescribing practices are essential to improve patient outcomes and preserve the effectiveness of antibiotics.

## Highlights

• 98% of patients received empirical antibiotics, though 68.3% of cultures were negative and many were mismatched.
• Resistance exceeded 50% for Ceftriaxone and Cefepime; Cefuroxime showed the lowest resistance at 42%.
• Correct empirical therapy improved prognosis (p=.029), while Ceftriaxone was linked to poorer outcomes (p=.017).
• Delayed culture results, often arriving after discharge, led to a heavy reliance on EAT and worse patient outcomes.
• Findings highlight an urgent need for better diagnostics and stewardship to tackle antibiotic resistance in Yemen.

## 1 Introduction

Antibiotics have revolutionized modern medicine by significantly reducing deaths and complications from bacterial infections [1]. However, their widespread and often inappropriate use has fueled the rise of antibiotic resistance, a major global health threat identified by the WHO and other global organizations [[2], [3], [4]]. This resistance occurs when bacteria adapt to resist the effects of drugs, making treatments less effective and increasing mortality [5,6]. The problem is more abundant in hospitals due to extensive use of broad-spectrum antibiotics for critically ill patients, contributing to the rise of multi-drug resistant organisms (MDROs) [[7], [8], [9], [10]].

This issue is complicated by the notable decline in the development of new antibiotics, largely due to economic and regulatory challenges and the limited duration of antibiotic treatments compared to drugs for chronic diseases. [[11], [12], [13]]. As a result, healthcare systems are extensively depended on the existing antibiotics, increasing the risk of bacterial resistance development. In low- and middle-income countries like Yemen, the challenge of antibiotic resistance is exacerbated by economic constraint, limiting access to diagnostic tools [[14], [15], [16]]. Tertiary hospitals in Sana'a, which provide advanced care to critically ill patients, face significant gaps in data on antibiotic use and resistance. The extensive use of broad-spectrum antibiotics in these settings even without bacteriological confirmation, coupled with inadequate diagnostic services and surveillance, further exacerbates the risk of resistance [[17], [18], [19], [20]].

This study aims to fill this gap by analyzing empirical antibiotic use and resistance patterns in a tertiary hospital in Sana'a. The findings will help inform better antibiotic stewardship policies, essential for preserving the effectiveness of existing drugs and improving patient outcomes, especially in resource-limited settings like Yemen. As the first study of its kind in Yemen, it also aims to serve as a model for future research and monitoring efforts in the region. The study's objectives include evaluating antibiotic use, dosing accuracy, and bacterial resistance, as well as assessing the impact of empirical antibiotic therapy on patient outcomes.

## 2 Methodology

### 2.1 Study population

The study population included adult patients admitted to the medical and surgical wards, which in this hospital, also includes sub-specialty wards like Obstetrics and Gynecology within the surgical division. These wards were divided into four distinct sections, encompassing both male and female patients across general and VIP wards3. (Note: Pediatrics was excluded based on the inclusion criteria for adult patients (≥18 years old) 4).

### 2.2 Study design

This study was designed as a prospective cross-sectional analysis to evaluate antibiotic use and resistance patterns among hospitalized patients. The cross-sectional approach was chosen to capture a detailed snapshot of antibiotic practices at a specific point in time.

### 2.3 Study location

The research took place at the University of Science and Technology Hospital (USTH) in Sana'a, Yemen's capital. Established in 2005, USTH is one of the region's largest private teaching hospitals, with a total bed capacity of 190. The hospital includes three critical care units: a 15-bed Intensive Care Unit (ICU), a 10-bed Cardiopulmonary Care Unit (CCU), and a 14-bed Neurological Intensive Care Unit (NICU). The study focused on the adult medical and surgical wards, which include 104 beds. All culture samples were collected, processed, and analyzed in the hospital's microbiology laboratory, where antibiotic susceptibility patterns were determined using the Kirby-Bauer disk diffusion method.

### 2.4 Study period

Data for this study were collected over a two-month period from January 12 to March 11, 2024.

### 2.5 Study sample

The study sample included all adult patients admitted to the medical and surgical wards who met the inclusion criteria during the study period.

After applying the inclusion and exclusion criteria, the final sample consisted of 101 cultures out of 188, representing 80 of the 133 patients. This method follows WHO guidance for point-prevalence surveys in hospitals with fewer than 500 beds, where complete enumeration is recommended instead of statistical sampling [21].

### 2.6 Inclusion and exclusion criteria

- **Inclusion Criteria:** All hospitalized adult patients in the surgical and medical wards who had a culture sample sent and received one or more systemic antibiotics on admission.
- **Exclusion Criteria:** Patients aged under 18 years, those without a culture test or antibiotic use, patients transferred from critical care units or from other hospitals, and patients on antifungal, antiviral, or anti-TB medications without antibiotics.

### 2.7 Study data

Data were collected by investigator at the patient level, including medical file number, sensitivity results for all antibiotics, type of sample (e.g., sputum, urine, blood, etc.), culture request and result dates, main diagnosis, comorbidities, infection monitoring parameters (C-reactive protein [CRP], white blood cell count [WBC], procalcitonin [PCT]), vital signs (blood pressure [BP], heart rate [HR], respiratory rate [RR], temperature [T]), and discharge status.

### 2.8 Data source

Data were collected manually from patient files and medication charts daily, usually between 8:00 a.m. and 2:00 p.m., with occasional extensions to ensure completeness. Culture data were obtained from the hospital's electronic system, as some results were not recorded in patients' files and often became available after patient's discharge. For patients discharged or admitted outside the standard collection hours, data were also retrieved electronically.

### 2.9 Data collection forms

A collection form was created to collect data on antibiotic use, susceptibility patterns, and clinical parameters, organized into four pages (cultures and patient info, antibiotic data, lab results and vital signs, and drug resistance risks).

### 2.10 Enhancing data accuracy and minimizing bias

To improve data accuracy and reduce biases, the following steps were implemented:

• A daily review of electronic health records (EHR) to monitor newly admitted patients.
• A double-check system in which two independent reviewers cross-verified the collected data against the manually entered data to detect and correct any errors or inconsistencies.

### 2.11 Data preparation

Data were first entered into a Microsoft Excel (2019) file and then cross-verified. After verification, the data were imported into SPSS for analysis.

### 2.12 Classifications

a. **Treatment Outcomes:** Classified based on changes in WBC, CRP, and vital signs tests from baseline to final results. Outcomes were categorized into three categories. **Good prognosis:** was determined if the final test results showed improvement compared to the baseline, regardless of whether the final results were normal or abnormal; **A Poor prognosis:** was indicated when the final results remained abnormal or worsened, or when one test result improved but did not return to normal. It was also classified as poor if the patient died, was transferred to the ICU, or was discharged against medical advice (DAMA) [22] due to treatment failure; and **Inapplicable category:** applied when the final and baseline results were normal, data were only available for one day, or if lab tests were not conducted.
b. **Empirical Antibiotic Appropriateness:** Antibiotics were classified as appropriate, inappropriate, or inapplicable based on sensitivity to cultured organisms **Appropriate**: was determined if at least one prescribed antibiotic showed sensitivity to the cultured organism, even if others did not, or if the antibiotic was not tested but was known to be effective based on guidelines like The Sanford [23] Guide to Antimicrobial Therapy. **Inappropriate**: was identified if all prescribed antibiotics showed resistance or if Candida was isolated. **Inapplicable**: when culture results were negative. The appropriate antibiotics were further categorized into “**Necessary**” or “**Unnecessary**” based on the isolated bacteria targeting. For example, an antibiotic was considered “**Unnecessary”** if it was sensitive to the cultured bacteria but the bacteria did not require that specific antibiotic, such as prescribing anti-pseudomonal agents for non-Pseudomonas Aeruginosa bacteria or using Vancomycin when MRSA was not isolated [24].
c. **Age Grouping**: Patients were grouped by age into four categories: young adult (18-25), adult (26-44), middle-aged (45-59), and old age (≥60) [25].

### 2.13 Measuring variables and metrics used

i. **Empiric Antibiotic Therapy (EAT):** Initial antibiotic treatment before pathogen identification.
ii. **Definitive Antibiotic Therapy (DAT):** Targeted treatment based on susceptibility testing.
iii. **Prevalence of Antibiotic Sensitivity (ABS%):** was calculated as the percentage of cultures sensitive to each antibiotic separately.

### 2.14 Data analysis

Data analysis was conducted using IBM SPSS Statistics for Windows, Version 27.0. Armonk, NY: IBM Corp. Descriptive statistics were reported as medians with interquartile ranges (IQR) for continuous nonparametric variables, and as percentages with absolute values for categorical variables. Bivariate correlation test was (with 95% CI) done to explore associations between variables. A crosstab analysis was performed to evaluate the association between treatment outcomes and antibiotics appropriateness, as well as other independent factors. Significant associations were identified and reported using 2-sided p-values from Chi-Square or Fisher's Exact Test. A p-value of < .05 was considered statistically significant.

## 3 Results

**Fig. 1:**
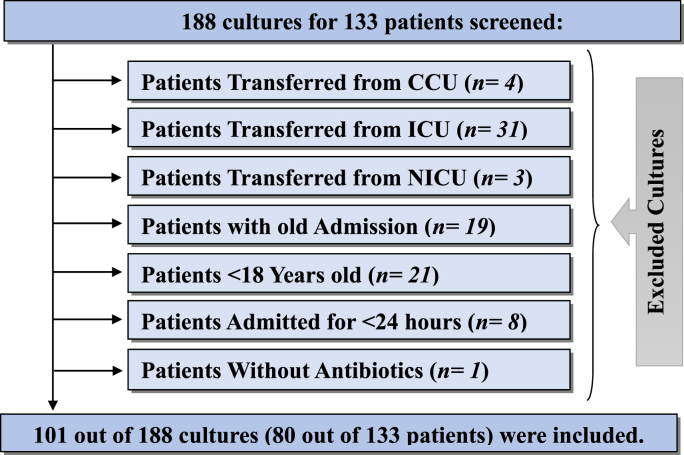
| *Case Selection Flowchart of participants Screened for analysis.* Flowchart of the patient and culture selection process. The diagram illustrates the screening of 188 cultures from 133 patients, leading to the final inclusion of 101 cultures from 80 unique patients based on specific inclusion and exclusion criteria. Major reasons for exclusion included age under 18 years (n = 21), transfer from critical care units (n = 38), and with old Admission (n = 19).

During the two-month study period, 101 cultures from 80 unique patients were included in this study. The demographic data of the patients and the characteristics of the cultures are summarized in Table 1, Table 2, and the selection flowchart of included patients is shown on Fig. 1. The mean age of the patients was 52.3 years (*SD* = 18.4), with 52.5% (42/80) being males. The average length of hospital stay was 4.4 days (*SD* = 3.6), and 58.8% (47/80) of the patients were admitted to VIP wards. The urine (28.7%) and blood (20.8%) were the most sources for cultures sampling and (57.4%, 58/101) of cultures were taken after antibiotics administration. The majority of patients (83.8%; 67/80) had a community-acquired infection and more than half of culture results (60.4%) appeared after patients’ discharge. Over two-thirds (68.3%; 69/101) of cultures were negative and (5%) were positive for Candida. About 51.5% (52/101) of culture results appeared after three days with a range of 1.6 to 11.2 days.

**Table 1:** **|***Demographic and Clinical Characteristics of the Included Patients (N = 80).*

		N	%	Mean	SD	Range		95% CI	
								Lower	Upper
Age Group				52.33	18.39	18	96	2.73	3.22
	(18-25 Y)	9	11.3%						
	(26-44 Y)	20	25.0%						
	(45-59 Y)	15	18.8%						
	(≥60 Y)	36	45.0%						
Length of Hospital Stays (LOS)				4.40	3.60	1	26	1.74	2.11
	1 to 2 days	26	32.5%						
	3 to 5 days	38	47.5%						
	6 to 10 days	12	15.0%						
	>10 days	4	5.0%						
Culture Duration (Days)				3.97	1.95	1.6	11.2	2.24	2.53
	1-2 Days	9	8.9%						
*(N = 101*)	>2 to 3 Days	40	39.6%						
	>3 Days	52	51.5%						
Gender	Male	42	52.5%					1.36	1.59
	Female	38	47.5%						
Ward	Male VIP	27	33.8%					2.10	2.62
(*N* = 80)	Male General	15	18.8%						
	Female VIP	20	25.0%						
	Female General	18	22.5%

**Table 2:** Microbiological profile: Culture source, timing, and pathogen distribution (N = 101).

		N	%	Overall Resistance %	95% CI	
					Lower	Upper
Culture Results	Negative	69	68.3%		.25	.48
	Positive For Bacteria	27	26.7%			
	Candida	5	5.0%			
Isolated Bacteria	*Escherichia coli*	5	18.5%	**56%**	1.69	3.34
	*Pseudomonas aeruginosa*	4	14.8%	43%		
(*N* = 27)	*Staphylococcus aureus*	3	11.1%	18%		
	*Coagulase negative staphylococci*	2	7.4%	36%		
	*Enterobacter cloacae*	2	7.4%	26%		
	*Klebsiella pneumoniae*	2	7.4%	38%		
	*Streptococcus pyogenes*	2	7.4%	21%		
	*Staphylococcus hominis*	2	7.4%	36%		
	*Burkholderia gladioli*	1	3.7%	**70%**		
	*Diphtheroids spp.*	1	3.7%	8%		
	*Enterococcus spp.*	1	3.7%	**80%**		
	*Staphylococcus hemolyticus*	1	3.7%	38%		
	*Stenotrophomonas maltophilia*	1	3.7%	**62%**		

Sample Timing	Before Antibiotics Use	43	42.6%		1.48	1.67
	After Antibiotics Use	58	57.4%			
Infection Type	Community-Acquired	67	83.8%		1.08	1.25
	Hospital-Acquired	13	16.2%			
Result Date	On Discharge Day	14	13.9%		1.32	1.61
	Before Discharge	26	25.7%			
	After Discharge	61	60.4%			

Sample Source	Urine	29	28.7%		4.63	5.49
	Blood	21	20.8%			
	Pleural Fluid	14	13.9%			
	Sputum	9	8.9%			
	Aspirated fluid	8	7.9%			
	Pus	7	6.9%			
	General	6	5.9%			
	Stool	4	4.0%			
	Wound Soap	2	2.0%			
	Ascitic fluid	1	1.0%

Out of 27 positive bacterial cultures, the most commonly isolated organisms were *Escherichia coli* (18.5%), *Pseudomonas aeruginosa* (14.8%), and *Staphylococcus aureus* (11.1%). The highest resistance rates were observed in *Enterococcus spp*. (80%), *Burkholderia gladioli* (70%), *Stenotrophomonas maltophilia* (62%), and *E. coli* (56%) (Table 3).

**Table 3:** **|***Primary Diagnoses, Comorbidities, and Clinical Outcomes of the Study Population.*

		N	%	95% CI	
				Lower	Upper
Main Diagnosis	Respiratory System	31	38.8%	8.22	9.83
	Genitourinary System	19	23.8%		
(*N* = 80)	Digestive System and Associated Organs	7	8.8%		
	Blood or Blood-Forming Organs	6	7.5%		
	Musculoskeletal System or Connective Tissue	6	7.5%		
	Neoplasms	5	6.3%		
	Diseases of Skin	2	2.5%		
	Others^a^	4	5%		

Number of Comorbidities	Undocumented	28	35%	.95	1.43
	One	24	30%		
	Two	18	22.5%		
	Three	8	10%		
	Four	2	2.5%		

Comorbidities	Cardiovascular-Diseases (CVD)	28	27%		
(*N* = 104)	Kidney Disease (Chronic, Acute, and ESRD)	20	19%		
	Diabetes Mellitus	17	16%		
	Chronic Respiratory Diseases (COPD, TB)	13	13%		
	Neoplasms or Post- Kidney Transplant	12	12%		
	Post-Cerebrovascular-Accident (CVA)	7	7%		
	Others^b^	7	7%		

Discharge status	Discharged by Doctor Order	71	88.8%	1.06	1.36
(*N* = 80)	Discharge-Against-Medical-Advice (DAMA)	4	5.0%		
	Transfer to Intensive-Care Units	2	2.5%		
	Death	3	3.8%		

Treatment Outcomes	Good Prognosis	33	41.3%	1.65	2.00
	Poor Prognosis	28	35.0%		
	Inapplicable	19	23.8%		
Antibiotics Appropriateness	Appropriate	19	18.8%	2.36	2.67
	Inappropriate	11	10.9%		
	Inapplicable (***or if Negative Cultures***)	71	70.3%		
Is Appropriate Necessary?	Yes	4	21.1%	1.59	1.99
	No	15	78.9%		

COPD=Chronic-Obstructive-Pulmonary-Disease; ESRD = End-Stage-Renal-Diseases; TB = Tuberculosis.

a Other diagnoses: Stroke (N = 1), Irregular menses (N = 1), Hyperkalemia (N = 1), and Febrile illness (N = 1).

b Other Comorbidities: Measles (N = 1), Hypokalemia (N = 1), Neuropathy (N = 1) Partial epilepsy (N = 1), Osteomyelitis (N = 1), Spinal-Instability (N = 1), and hypothyroidism (N = 1).

**Fig. 2:**
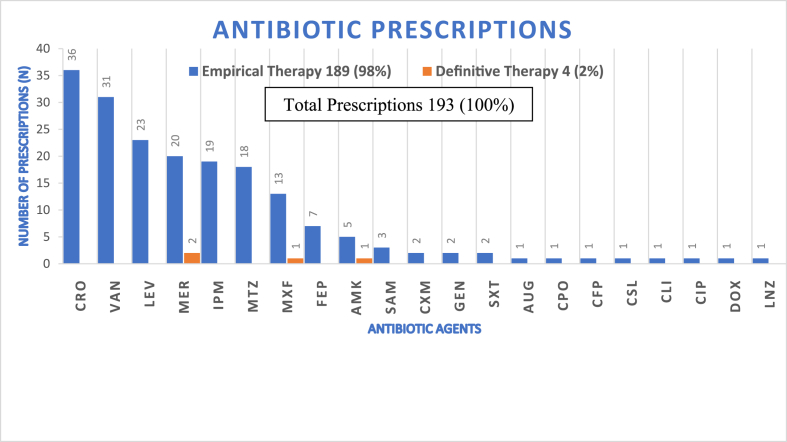
**|***Frequency and distribution of antibiotic prescriptions (N = 193).* The stacked bars distinguish between empirical and definitive therapy, highlighting a high reliance on empirical treatment (98%). Abbreviations follow the WHONET antimicrobial coding system: **CRO**: Ceftriaxone; **VAN**: Vancomycin; **LEV**: Levofloxacin; **MER**: Meropenem; **IPM**: Imipenem/Cilastatin; **MTZ**: Metronidazole; **MXF**: Moxifloxacin; **FEP**: Cefepime; **AMK**: Amikacin; **SAM**: Ampicillin/Sulbactam; **CXM**: Cefuroxime; **GEN**: Gentamicin; **SXT**: Co-trimoxazole; **AUG**: Amoxicillin/Clavulanic acid; **CPO**: Cefpirome; **CFP**: Cefoperazone; **CSL**: Ceftriaxone/sulbactam; **CLI**: Clindamycin; **CIP**: Ciprofloxacin; **DOX**: Doxycycline; and **LNZ**: Linezolid. (The total prescriptions for each antibiotic [Empirical & Definitive])/Total prescriptions for all antibiotics [Empirical & Definitive]) X 100.

The most frequent diagnoses were respiratory system diseases (38.8%; 31/80), followed by genitourinary diseases (23.8%; 19/80) and digestive system disorders (8.8%). A significant proportion of patients (35%; 28/80) had no documented comorbidities, while 30% had one comorbidity, 22.5% had two, 10% had three, and 2.5% had four comorbidities. The most common comorbidities included cardiovascular diseases (27%; 28/104), chronic kidney disease (19%; 20/104), diabetes mellitus (16%; 17/104), and chronic respiratory diseases (13%). About 88.8% (71/80) of patients were discharged by physician's order. Treatment outcomes indicated a good prognosis in 41.3% (33/80) of cases and a poor prognosis in 35% (28/80) of cases. Approximately 18.8% (19/101) of empirical antibiotic prescriptions were appropriate, with 21.1% classified as appropriate-necessary and 78.9% as unnecessary (Fig. 2).

A total of 21 different antibiotics were prescribed empirically, accounting for 98% (189/193) of prescriptions, while three antibiotics were prescribed as definitive therapy after culture results were obtained (4 prescriptions). The most commonly prescribed antibiotics, representing two-thirds of the total prescriptions, were: Ceftriaxone (18.7%), Vancomycin (16.1%), Levofloxacin (11.9%), Meropenem (11.4%), Imipenem/Cilastatin (9.8%), and Metronidazole (9.3%). Meropenem was prescribed twice as definitive therapy, while Moxifloxacin and Amikacin were each prescribed once (Fig. 2).

**Fig. 3:**
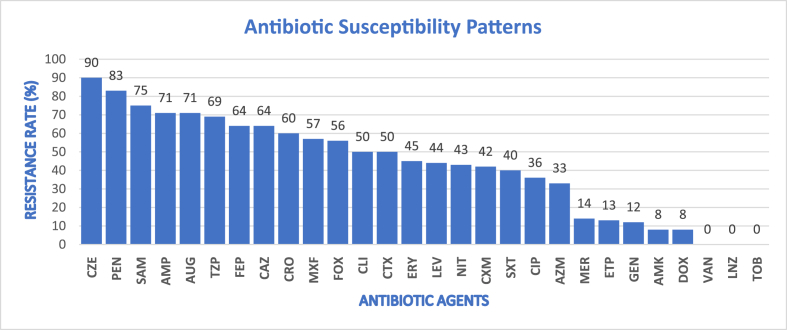
**|***Descriptive Statistics of Antibiotic Susceptibility Patterns.* Antibiotic resistance patterns of isolated bacterial pathogens. Values represent the percentage of resistance ($R\%$) for each tested agent. Abbreviations are based on WHONET antimicrobial codes: **CZE**: Cefazolin; **PEN**: Penicillin; **SAM**: Ampicillin/Sulbactam; **AMP**: Ampicillin; **AUG**: Amoxicillin/Clavulanic acid; **TZP**: Piperacillin/Tazobactam; **FEP**: Cefepime; **CAZ**: Ceftazidime; **CRO**: Ceftriaxone; **MXF**: Moxifloxacin; **FOX**: Cefoxitin; **CLI**: Clindamycin; **CTX**: Cefotaxime; **ERY**: Erythromycin; **LEV**: Levofloxacin; **NIT**: Nitrofurantoin; **CXM**: Cefuroxime; **SXT**: Co-Trimoxazole; **CIP**: Ciprofloxacin; **AZM**: Azithromycin; **MER**: Meropenem; **ETP**: Ertapenem; **GEN**: Gentamicin; **AMK**: Amikacin; **DOX**: Doxycycline; **VAN**: Vancomycin; **LNZ**: Linezolid; and **TOB**: Tobramycin.

With the exception of Cefuroxime, which had a resistance rate of 42%, overall resistance to all tested Beta-lactams exceeded 50%. Cefazolin exhibited the highest resistance rate at 90%. Resistance rates for Ampicillin, Ampicillin/Sulbactam, and Amoxicillin/Clavulanic acid were all above 70%. Additionally, Moxifloxacin, Piperacillin/Tazobactam, and Cefepime showed resistance rates of 57%, 69%, and 64%, respectively (Fig. 3).

The appropriateness of EAT significantly influenced treatment outcomes. All patients (100%; 11/11) who were discharged with a good prognosis received appropriate empirical antibiotic therapy, while 50% of patients discharged with a poor prognosis had received inappropriate empirical antibiotics (*p* = .029).

The number of antibiotics used per patient also had a significant impact on treatment outcomes. A good prognosis was observed in 87.9% of patients who received two or three antibiotics, compared to 0% of patients who received only one antibiotic and 12.1% of patients who received more than three antibiotics (*p* = .005).

Ceftriaxone use was found to inversely affect treatment outcomes. Patients received Ceftriaxone were more likely to have a poor prognosis (62.96%; 17/27), while 69.7% (23/33) of patients with a good prognosis did not receive Ceftriaxone, *X*^*2*^ (1, *N* = 61) = 5.68, *p* = .017.

Associations were analyzed between treatment outcomes and the timing of culture results as well as the type of infection (community-acquired *vs*. hospital-acquired). Discharging patients before culture results became available was associated with a poor prognosis in 78.6% of cases in compare to 21.4% in those who received the culture results while they are on hospital, *X*^*2*^ (1, *N* = 61) = 3.87, *p* < .049. Although not statistically significant, a higher proportion of patients with community-acquired infections (81.8%) had a good prognosis compared to those with hospital-acquired infections (18.2%), *X*^*2*^ (1, *N* = 61) = .52, *p* = .469 (Table 4).

**Table 4:** **|***Statistical Association Between Antibiotic Appropriateness, Treatment Variables, and Patient Prognosis.*

		Treatment Outcomes (Prognosis)					
		Good	Poor	Total			
		N (%)	N (%)	N (%)	Value	df	P^a^
Antibiotics Appropriateness	Yes	11 (100%)	3 (50.0%)	14 (82.4%)			.029^b^
	No	0 (.0%)	3 (50.0%)	3 (17.6%)			
	Total	11 (100%)	6 (100%)	17 (100%)			
Number of Antibiotics Used	1	0 (.0%)	6 (21.4%)	6 (9.8%)			.005^b^
	2 - 3	29 (87.9%)	16 (57.1%)	45 (73.8%)			
	≥4	4 (12.1%)	6 (21.4%)	10 (16.4%)			
	Total	33 (100%)	28 (100%)	61 (100%)			

Ceftriaxone Prescription	Yes	10 (30.3%)	17 (60.7%)	27 (44.3%)	5.68	1	.017^c^
	No	23 (69.7%)	11 (39.3%)	34 (55.7%)			
	Total	33 (100%)	28 (100%)	61 (100%)			
Result Date (*Before/After Discharge*)	Before	15 (45.5%)	6 (21.4%)	21 (34.4%)	3.87	1	.049^c^
	After	18 (54.5%)	22 (78.6%)	40 (65.6%)			
	Total	33 (100%)	28 (100%)	61 (100%)			
Infection Type	Community	27 (81.8%)	21 (75.0%)	48 (78.7%)	.52	1	.469^c^
	Hospital	6 (18.2%)	7 (25.0%)	13 (21.3%)			
	Total	33 (100%)	28 (100%)	61 (100%)			

a 2-Sides P-Value.

b Fisher's Exact Test.

c Chi-Square Test.

The negative culture results were equally distributed between good and poor prognoses (40% each), with a smaller proportion in the inapplicable category, meaning that there is no statistically significant association between negative culture results and treatment outcomes (Fisher's Exact *p* = .285). Additionally, while most patients with negative results (52.7%, 29/55) were associated with longer hospital stays (>3 days), this trend is not statistically significant (*p* = .336). Furthermore, a higher proportion of negative culture results (58.0%, 40/69) were obtained after antibiotic administration compared to before (42.0%, 29/69), but similar trends were observed for positive bacterial cultures and Candida, yet no significant difference was found between culture results before and after antibiotic administration (*p* = .999) (Table 5).

**Table 5:** **|***Correlation of Culture Results with Treatment Outcomes, Hospital Stay, and Antibiotic Timing.*

		Culture Results			Total	
		Negative	Positive	Candida		
		N (%)	N (%)	N (%)	N (%)	P^a^
Treatment Outcomes (Prognosis)	Good	22 (40.0%)	11 (47.8%)	0 (.0%)	33 (41.3%)	.285
	Poor	22 (40.0%)	5 (21.7%)	1 (50.0%)	28 (35.0%)	
	Inapplicable	11 (20.0%)	7 (30.4%)	1 (50.0%)	19 (23.8%)	
Total		55 (100.0%)	23 (100.0%)	2 (100.0%)	80 (100.0%)	
Length of Hospital Stays	<3 Days	18 (32.7%)	7 (30.4%)	1 (50.0%)	26 (32.5%)	.336
	3 Days	8 (14.5%)	6 (26.1%)	1 (50.0%)	15 (18.8%)	
	>3 Days	29 (52.7%)	10 (43.5%)	0 (.0%)	39 (48.8%)	
Total		55 (100.0%)	23 (100.0%)	2 (100.0%)	80 (100.0%)	
Sample Timing	Before ABs	29 (42.0%)	12 (44.4%)	2 (40.0%)	43 (42.6%)	.999
	After ABs	40 (58.0%)	15 (55.6%)	3 (60.0%)	58 (57.4%)	
Total		69 (100.0%)	27 (100.0%)	5 (100.0%)	101 (100.0%)	

a Fisher Exact Test Sig. (2-Sided). **Abs**: Antibiotics.

## 4 Discussion

To my knowledge, this study is the first of its kind in Yemen to evaluate empirical antibiotic use, and susceptibility patterns specifically in hospital wards rather than ICU settings. Key findings include: a high proportion of empirical antibiotic therapy (98%) with notable high utilization of Beta-lactams; a significant rate of negative culture results, and alarming high resistance rates to commonly used antibiotics such as Beta-lactams and Moxifloxacin; and associations between inappropriate antibiotic use and adverse outcomes, including longer hospital stays. These findings point to systemic issues in empirical antibiotic prescribing practices and diagnostic capabilities within the hospital, which have important implications for patient outcomes and the development of AMR.

A key challenge in this study was the high proportion of negative culture results, which significantly reduced the sample size available for analysis. Negative culture results can occur due to several factors, including prior antibiotic use before culture sampling, inadequate sample collection or handling, and the presence of non-culturable pathogens. These factors are likely exacerbated in resource-limited settings, further complicating the interpretation of empirical antibiotic use and resistance patterns [[26], [27], [28]].

The study also highlights the deviation from established guidelines, such as those provided by the World Health Organization (WHO) [4], particularly in the high rate of empirical antibiotics use. This practice not only increases the risk of AMR but also negatively impacts patient outcomes, including prolonged hospital stays and increased healthcare costs. Similar challenges have been reported in other studies, where inappropriate antibiotic use has been linked to increased resistance rates and poorer clinical outcomes [2,29,30].

The high resistance rates, exceeding 50%, were observed against most commonly used Beta-lactam antibiotics and other agents. This finding aligns with global reports that associate antibiotic misuse with rising resistance, particularly in hospital settings [31]. Furthermore, hospital-based studies have also linked the use of broad-spectrum antibiotics with increased resistance, underscoring the need for improved antibiotic stewardship and strong surveillance systems to monitor resistance patterns [8,32,33]. Promoting rational prescribing practices through audits, feedback, and decision-support tools is essential to optimize antibiotic use.

Appropriate empirical antibiotic therapy significantly improved treatment outcomes, with all patients receiving appropriate therapy being discharged with a good prognosis. In contrast, inappropriate therapy was linked to poor outcomes in 50% of cases, consistent with existing literature [[34], [35], [36]]. The use of two or three antibiotics was associated with better outcomes compared to monotherapy or the use of more than three antibiotics, echoing findings from other studies on combination therapy [37,38]. This emphasizes the importance of improving rapid diagnostic services to guide targeted antibiotic therapy effectively.

### 4.1 Study limitations and strengths

Several limitations of this study should be noted:

• **Single-Center Design:** The results may not be broadly applicable to other hospitals in Yemen or internationally due to variations in healthcare systems and antibiotic prescribing practices. However, since many physicians in Sana'a work across multiple hospitals and share similar practices, the findings are likely reflective of broader trends within the city.
• **Small Sample Size:** The study's limited patient and culture sample size may reduce the generalizability of the resistance patterns observed, so these findings should be interpreted with caution. Nevertheless, the use of culture results to evaluate the appropriateness of empirical antibiotics is well-justified, and the study provides important preliminary data, particularly in Yemen, where such research is rare.
• **Manual Data Collection:** Although manual data collection could introduce bias, measures such as independent cross-checking were implemented to minimize this risk.

Despite these limitations, this prospective study provides valuable insights into antibiotic use and resistance patterns in a tertiary hospital in Sana'a, Yemen. It contributes meaningfully to local and regional efforts to address antimicrobial resistance and improve antibiotic stewardship.

## 5 Conclusion

This study provides critical insights into the patterns of empirical antibiotic use, and susceptibility patterns in a tertiary hospital in Sana'a. Key findings include significantly high resistance rates to commonly used antibiotics. The most commonly prescribed antibiotics empirically were Ceftriaxone, Vancomycin, Levofloxacin, Meropenem, Imipenem/Cilastatin, and Metronidazole. The empirical use of antibiotics often did not align with susceptibility patterns, leading to poor clinical outcomes. Additionally, the high proportion of negative cultures highlights the limitations of the current diagnostic capabilities and the potential overuse of empirical antibiotics. To address these challenges, comprehensive antibiotic stewardship programs should be implemented. These programs should focus on regular training and education for healthcare professionals, standardized protocols for empirical therapy, and enhanced surveillance systems. Furthermore, promoting rational prescribing practices and improving rapid diagnostic services are crucial for optimizing antibiotic use and combating antimicrobial resistance.

**Recommendation for Future Research:** Future research should expand on these findings through multi-center studies to increase generalizability across Yemen's tertiary hospitals. Furthermore, longitudinal surveillance focusing on the economic impact of inappropriate EAT and intervention studies evaluating the effectiveness of new antimicrobial stewardship programs are warranted to support national policy changes.

## CRediT authorship contribution statement

**Adel Alshaikh:** Writing – review & editing, Writing – original draft, Visualization, Validation, Software, Resources, Project administration, Methodology, Investigation, Formal analysis, Data curation, Conceptualization. **Mohammed Kubas:** Supervision, Project administration, Methodology, Conceptualization.

## Ethical approval

The study ensured strict confidentiality of patient information. Since no personal data were collected, patient consent was not required. Data access was limited to the investigator and supervisor. The research ethical approval (no. LIU/UREC/2024/001) obtained from the Lebanese International University (LIU) University Research Ethics Committee (UREC), and permission for data collection was granted by the medical administration of USTH.

## Declaration of generative AI and AI-assisted technologies in the writing process

During the preparation of this work the author used ChatGPT in order to help improving readability by checking spell and grammars. After using this tool, the author reviewed and edited the content as needed and takes full responsibility for the content of the publication.

## Funding

None.

## Declaration of competing interest

The authors declare that they have no known competing financial interests or personal relationships that could have appeared to influence the work reported in this paper.
